# Beyond Boundaries and Slippery Slopes: A Case for the Therapeutic Benefits of Gift-Giving in Psychotherapy

**DOI:** 10.7759/cureus.71354

**Published:** 2024-10-13

**Authors:** Sheetal Lakhani, Naveen Grover, Vishal Dhiman

**Affiliations:** 1 Department of Psychology, Vidyashilp University, Bengaluru, IND; 2 Department of Clinical Psychology, Institute of Human Behaviour and Allied Sciences (IHBAS), New Delhi, IND; 3 Department of Psychiatry, All India Institute of Medical Sciences Rishikesh, Rishikesh, IND

**Keywords:** boundaries, chronic schizophrenia, ethics, gifting, psychotherapy, therapeutic alliance

## Abstract

Clients with chronic and severe psychiatric illnesses, such as schizophrenia, often present with significant challenges in psychotherapy, particularly in establishing a stable therapeutic alliance. This paper details a novice therapist’s experience and initial challenges in establishing a bond with a client diagnosed with chronic schizophrenia. After initial efforts and 12 months of fluctuating client engagement, the treatment team recognized the need for a stronger alliance. Unconventional methods, including the strategic use of ethical gift-giving, were explored to foster the alliance with the client, which eventually led to improved client engagement and therapeutic results. The paper critically reviews the existing literature on the ethics of gifting in psychotherapy, highlighting the scarcity of research on the therapeutic use of gifts and the predominant cautionary stance in ethical codes with respect to gifting. We argue for the potential benefits of, and a proposed framework for, ethical gifting when properly executed, particularly for clients in the concrete operational stages of cognition and impaired social and interpersonal functioning. The framework underscores factors such as formulation, cultural context, and therapeutic motive, emphasizing the importance of supervision and consultation. Insights from the trainee therapist's reflections shed light on the process, revealing initial reservations followed by gradual acceptance and professional development as a reflective therapist. These reflections underscore the significance of incorporating reflective practice in psychotherapy, especially when navigating challenges and dilemmas. We advocate further empirical research on the role and implications of gifting in psychotherapy.

## Introduction

Clients with chronic and severe psychiatric illnesses often present with significant challenges in psychotherapy. In some individuals with chronic schizophrenia, for example, positive and negative symptoms can complicate engagement in long-term therapeutic interventions due to slow progress, socio-occupational functioning difficulties, and lack of social support, as well as a lack of readily visible gains from the intervention. Additionally, the fluctuating nature of symptoms in schizophrenia may pose ongoing challenges to maintaining a consistent therapeutic alliance. Especially if clients are in the concrete operational mode of cognitive functioning and there are difficulties with object relations, usual alliance-building strategies such as reassurance, warmth, and others [1] may not be sufficient to strengthen the alliance.

Establishing a therapeutic alliance with clients with complex presentations may be even more troublesome for beginning therapists, who may struggle with a myriad of problems in this crucial period of their professional development. These challenges include high anxiety concerning performance, insufficient conceptual or technical knowledge, high expectations from the self, and unrealistic idealization of the therapy process [2]. The high emotional involvement of the client and the counsellor and the need to ensure a professional therapeutic relationship result in ethical dilemmas and conflicts in psychotherapy, another challenge for beginning psychotherapists. Codes of ethics such as the American Counselling Association code of ethics [3] are available as valuable guidance for psychotherapists but may not be sufficiently specific to address the nuances of each specific case.

On the other hand, cases involving unique challenges could serve as valuable opportunities for training novice therapists to navigate difficult situations in therapy. This paper examines the training and reflective experience of one such case study involving unique challenges that led to a valuable opportunity for the training of a novice therapist. Due to the complex case presentation (elaborated in the case presentation section) and the failure of conventional alliance-building strategies, gift-giving from the therapist to the client was considered an experimental method to enhance the therapeutic alliance. The rationale has been elaborated in the current paper after reviewing the literature on gifting and ethical codes.

Gifting is one of the most controversial ethical dilemmas in psychotherapy, with apprehensions about blurring boundaries, dual relationships, and potential harm/impact on the client and the therapeutic alliance [4]. Most of the literature discusses the considerations in receiving gifts from the client to the therapist. For instance, standard A.10.e. of the American Counselling Association code of ethics includes a paragraph on ethical considerations while receiving gifts from clients but not on giving gifts to clients [3]. The standard suggests counsellors consider the therapeutic relationship, gift value, client's intention, and their own motives when deciding whether to accept a gift. The California Association of Marriage and Family Therapists Code of Ethics also emphasizes understanding the clinical and cultural implications of gifts, their value and impact on the therapeutic relationship, and the motivations behind giving, receiving, or declining gifts by both clients and therapists [5]. The Behaviour Analyst Certification Board (BACB code) maintains that gifts should be avoided due to the possibility of venturing into multiple relationships and categorically sets the financial limit at 10 U.S. dollars [6]. However, it is important to note that while ethical codes primarily focus on the risks associated with gifts, they do not prohibit therapists from accepting or receiving gifts.

The act of presenting a gift to the client is a rare or rarely discussed event, as evidenced by a survey of the existing literature. Almost no empirical work has been done on this matter previously, and the codes mention the same rules for gift giving as gift receiving, which seem to be majorly based on the empirical work on receiving gifts from clients. However, it is clear that it is considered an area of ethical greyness and even openly frowned upon by some thinkers, especially in the psychodynamic school [4]. The ghosts of the old slippery slope argument [7] in ethics, which argues that one ethical transgression would definitely result in a serious ethical violation, may still be active in the commonly followed method of fearing ethical boundary violations in therapy, resulting in the therapist being on guard and conservative with matters that are considered ethically grey.

Knox [4] discusses the work of Langs [8] who insist that therapists should never give their clients tangible gifts except for children. The limited discussion available on gift-giving is mostly in the domain of child psychotherapy, such as play therapy [9], and advocates giving small, inexpensive gifts to help establish the therapeutic relationship, reinforce progress, let the client know that they are liked, or serve as transitional objects. Gifting children in therapy to strengthen the therapeutic alliance is based on the notion that children may engage with therapists in tangible ways [10]. Nonetheless, this approach is met with notable skepticism among clinicians. Knox discusses the work of Gutheil and Gabbard [4], who opposes the act of gifting children in play therapy, propagating that it is not right to “buy” the cooperation of a child.

As for adult clients, some psychodynamic theorists opine that gifts from clients are indicators of transference and accepting gifts is a transgressing of boundaries or the “frame” of the relationship [4]. One could then extrapolate that gifting the client would be an unforgivable transgression of the therapeutic frame, as it would encourage countertransference and transference in a relationship. An interesting anecdote of the psychoanalytic narrative on the dire consequences of gifting the client has been elaborated by Knox [4], wherein after Freud [11] gifted his client Rat Man, his progress in his therapeutic work was impaired. Of course, one would have to conduct more studies to understand and replicate the idea and to implicate causation, i.e., that work with Rat Man was impaired due to the gifting process. One would also have to consider that in the psychoanalytic mode of therapy, the therapist is supposed to be "abstinent,” as Freud suggested [12], and thoughts about the role of the therapist have evolved since then. Another prevalent concern is that excessive gift-giving might exert undue influence on the client, promote dependency on the therapist, or enable the therapist to misuse the act of gifting for personal advantage [9]. We propose that such views cast doubt on the professionalism and judgement of individual therapists, questioning their capacity to make sound therapeutic decisions based on their training, clinical experience, and discretion.

There are infrequent instances of a more open stance towards gifting in contemporary psychotherapies. Therapist gifts in adult psychotherapy include the gift of self-disclosure, non-sexual touch, extra time, and the gift of presence [13]. A seminal paper by Chused [14] highlights the limitations of verbal interventions, especially with individuals in the concrete operations stage. The excellent argument made by Chused is that clients may not always be capable of “hearing or understanding the words of the therapist.” Levin and Wermer [12] have suggested that in work with children, the initial phase is marked by a lot of “giving” from the therapist to achieve a satisfactory level of alliance. Meares and Anderson [15] argue that gifts may serve the functions of transitional objects in psychotherapy. Interestingly, multiple instances of giving different types of gifts, from the gift of time and presence to the gift of an allowance per week, have been elaborated in the psychoanalytic literature, with some reporting improvement in task mastery, ego development, and symptom reduction [12]. It is noteworthy that there are reports that Freud would often send various gifts to his clients, thus discrediting the staunch opposition to gifting through the example of Freud [16]. These arguments can be extrapolated to adult clients [10], albeit with some caution and consideration. Chused usefully argues that it is the responsibility to engage in concrete actions to facilitate the communication of the alliance. Tangible gifts can also be equated to reinforcement in behavioural therapy or to the abstract gifts of extra time, for instance, that a therapist may be gifting a client. Hundert [17] asserted that inexpensive gifts may be given to children and “regressed” adults for a therapeutic purpose. Unfortunately, there is a lack of understanding as to how clients perceive such gifts, and there are very few empirical studies or anecdotal evidence from which to draw.

Unethical gift-giving from the therapist can be summarized as a gift given without enough consideration of the benefits and the harms as per the client's case formulation. It is important to consider here that ethical or unethical gift-giving can be detrimental to the therapeutic relationship and the progress of the client. Potential harm can come from the blurring of boundaries in the therapeutic relationship. Client dependence on the therapist or transference, as well as the possibility of ruptures in the therapeutic alliance, cannot be ignored. However, it may also be important to consider that all therapeutic events facilitate the formation of an alliance, including therapeutic ruptures [4].

In summary, there is very little literature on gifting the client, and it is mostly discouraging. It would, therefore, be an unpopular opinion to suggest the strategic and ethical use of culturally appropriate gifting to strengthen alliances and improve therapeutic outcomes. The authors argue that there could be some ways in which one could engage in gifts in psychotherapy without violating any ethical principles or codes of conduct. When executed appropriately and ethically, such practices could also serve as an effective strategy for engaging clients with complex presentations. For the purposes of the current study, a case study is presented, considering it is an advantageous methodology for selected, complex situations. We also summarize a framework for considerations in gifting and advocate more research to clarify the consequences of gifting following the case study. The current paper aims to begin a more open discourse on the strictness surrounding ethics in psychotherapy, especially in the matter of gifting.

The authors highlight a case study of therapy with a client suffering from chronic schizophrenia and a novice therapist. We zoom in on the experience of a beginning trainee therapist who saw the client for 100+ sessions over a span of two years. The therapy process had multiple goals, all of which required the client's regular engagement. Difficulties in engaging the client using conventional methods are described, along with the rationale for considering gift-giving. As part of the process, we used the unconventional method of gifting to strengthen the therapeutic alliance with the client. Careful consideration was given to the potential side effects of the alliance-building process employed. In cases of increased dependence on the therapist or other therapeutic ruptures, plans were made to work through the problems when they arose.

## Case presentation

The client was an unmarried and unemployed female in her early 30s and had had a long history of paranoid schizophrenia with comorbid obsessive-compulsive disorder and major depression. She was on ongoing psychiatric treatment for more than 10 years. The high dose of medicines resulted in side effects to a certain extent, further impacting the client’s functioning. Her prognosis was assessed to be poor as she had been stabilized on her maximum treatment doses. There were periodic bouts of psychotic symptoms and poor medication compliance with a high tendency to relapse. The client was diagnosed with treatment-resistant schizophrenia.

Her socio-occupational functioning was largely impaired. Clinical assessment revealed limited cognitive capacities due to long-term psychiatric illness and difficulties in social skills due to negative symptoms of the disorder. The presentation included concrete thinking and reduced capacity for abstract reasoning, the inability to follow an activity schedule, to remember to take the medicines regularly, interpersonal difficulties with parents (high burnout and expressed emotion in mother), reduced eye contact, speaking in a child-like tone, passive-aggressive or aggressive behaviour and lack of assertiveness, difficulties in social interactions with individuals from the community, lethargy, and other side-effects due to medicines. This client was unable to function and, therefore, had to stop working while she was training to be a teacher. Besides her psychiatrist and psychotherapists, the client was seeing her occupational therapist, all in the tertiary hospital setting. She often expressed frustration with the long-term nature of the treatment and the fact that she did not have “a stable life just like everyone else.” As a result, the client would frequently disengage from psychotherapy and occupational therapy and displayed signs of burnout with the chronic nature of the treatment. Lastly, the only social support the client had was from her father, who was also seen to frequent the hospital with her, often appearing fatigued and sometimes hopeless. In essence, the client was assessed to have difficulties in almost all areas of socio-occupational functioning and a symptomatic presentation.

Besides the above-mentioned difficulties, the treating team encountered a couple of unique challenges within the context of the setting and this particular case. The treatment setting was a tertiary hospital and a training institute in a metropolitan city in northern India. Trainee therapists completed their training every two years and were typically assigned therapy cases only in their second year of training, resulting in the client experiencing multiple therapist changes. However, the senior consultant psychologist (second author) for the case is the same for the client, and the handover usually involves a regular, standard process of written notes and sometimes joint meetings with the old and the new therapist. While it has sometimes been seen as advantageous for the clients, the change in trainee therapists posed an additional threat to the formation of a stable alliance with the current client. It may also be important to consider that the public hospital setting in India is usually very crowded, and to see a psychiatrist, the client and her father would often have to wait a few hours for her follow-up. The treating team had attempted to coordinate with the psychiatry department to reduce the client’s waiting time for such consultations. Nevertheless, one may be able to empathize with what seems to be a chronic institutionalization of the client, albeit on an outpatient basis. The current trainee therapist, a 25-year-old female at the time, had just begun her training in clinical psychology post-masters. She was assigned the client immediately upon joining her two-year-long course to combat the problem of frequent changes in therapists every year. The therapist was new to seeing clients for psychotherapy and nervous about her performance as a therapist. The supervisor of the trainee therapist was an experienced male professor at the university who had supervised all the previous therapists for the past six-plus years and met the client and her family periodically. Focused supervision, regularized group supervision meetings, peer supervision, academic discussions of ethical frameworks, and role play-based skills training, among other mechanisms, supported the trainee therapist throughout the journey.

At the onset of therapy with the current trainee therapist, the client explicitly expressed the challenges of building rapport with a new therapist each year. This was accompanied by a cautious and resistant demeanour in the client’s communication with the trainee therapist. Aggressive remarks from the client were quite tricky for the beginning therapist to manage. There were instances where the client would stop talking in the middle of the session and ask paranoid questions such as “Why are you looking at me like that?.” She would ask the trainee therapist if she was mocking her or plotting against her along with other members of the treating team. Once, she picked up her bag and abruptly left the session. After such an occurrence, the client typically missed a couple of sessions or turned up as if nothing had happened in the previous session. However, gradually, she started apologizing to the therapist after such events, explaining sometimes how she had not taken her medicines on those occasions. The client would also ask personal questions of the therapist and bring up the therapist’s clothes or accessories in the session to appreciate them, making it more challenging for the beginning therapist to strategize the alliance building. It was assessed that the client showed interest in concrete engagement with the therapist.

Efforts were made to engage the client using various conventional methods of alliance-building. The trainee therapist dedicated parts of the session to discussing the tasks and goals that the client found important. A strength-based approach was adopted in the therapy towards the client, enhancing her autonomy and self-esteem. Negotiation, an important resolution for alliance difficulties, was often employed, often seeming to be successful. However, within a period of time, the client would go back to her pattern of disengagement in psychotherapy.

Additionally, the goals for the therapy with the client were also ambitious. Based on the reviewed literature on schizophrenia interventions, a massed practice of social skills training was planned with the client to improve her engagement in the community. This was planned in an intensive outpatient therapy format with a frequency of three per week, coordinated with her occupational therapy sessions. It was seen that when the client stopped coming to the clinic, the symptoms worsened for her, resulting in conflicts at the family level and expressed emotions from family members. This would adversely impact her social-occupational functioning as well as her compliance with medication. This, in turn, hindered progress in therapeutic work, perpetuating a cycle of prolonged help-seeking with little or no visible improvement.

To facilitate concrete, tangible reinforcement for therapeutic engagement, tangible and intangible gifts were planned for the client. This was done keeping in mind the chronic course and nature of the client’s illness and treatment, the poor socio-occupational functioning and support, including pervasive feelings of rejection, the chronic institutionalization, and the client’s limited abstract reasoning and cognitive capacities. An intangible gift of accompanying the client to her occupational therapy sessions three times a week was also added, and some psychotherapy sessions were conducted after the OT sessions in that space. This was done based on the idea that an increased frequency of sessions improves the therapeutic alliance and also reduces the load on the client. The act of gifting was seen as a symbolic method of communicating unconditional regard and acceptance of the client in a language that was more understandable for the client. Potential far-reaching benefits and costs for the client and therapeutic relationship were considered, and management plans were developed for the potential problems that may arise from the gifting. For instance, the possibility of increased dependence on the therapist or therapeutic ruptures was anticipated, and we planned to address such occurrences using established therapeutic strategies typically employed in cases of client transference.

What was done and how?

After a year of therapy, the therapeutic bond continued to be weak and was determined to be inadequate to move forward in the therapeutic work. After a supervisory discussion and much thought, the trainee therapist provided the client with an inexpensive, small gift in the form of earrings (accessories the therapist appreciated) to facilitate the bond. This was done in the second year of therapy with the client before initiating the social skills training. The client appreciated the gift and gave positive feedback about it when asked. Additionally, three times a week, the therapist accompanied the client to her occupational therapy, a naturalistic setting where she was involved in artwork, such as candle-making and painting. Thus, a tangible and an intangible gift (time) was given to the client well into the therapeutic journey.

Following the gifts, in terms of the therapeutic alliance, it was seen that the cautious engagement style of the client quickly changed to noticeably increased interest and involvement in the therapy process and with the therapist. Her absences and missed appointments reduced, as did the frequency of paranoid behaviours towards the client. The client reported looking forward to the therapeutic work, and her adherence to homework and therapeutic tasks increased. Along with an improved quality of therapeutic alliance, the client was seen to be increasingly engaged in occupational and psychotherapy. These improvements were assessed by the treating team through clinical observation and mental status examinations.

Social skills training was initiated as she began coming regularly to her sessions, and role plays were conducted. She demonstrated increased comfort with the therapist, even in situations that previously elicited awkward behavior. Even though the client initially resisted the idea of meeting three times a week, she seemed to be more comfortable with the idea as the social skills training continued. The therapist was also starting to feel more at ease with the idea of meeting the client three times a week. The client’s compliance with medication also increased, and her family members also reported the same.

As the therapy setting was now a safe space for the client, and she was exhibiting comfort with the current therapist, another colleague with a friendly approach was added to the sessions on social skills training to further boost the challenge and assess the client’s confidence. A bond was seen to quickly develop with the new role-play participant, and the client was observed to be increasingly engaged in therapy. Around this time, her family members also reported that she was much more active and interactive. The social skills training focused on non-verbal skills: eye contact and facial expressions, talking in a childlike tone and verbally phrasing requests and saying no, talking to strangers, and making new friends. The idea of shame came up when the client shared that she did not know how to answer questions about what she was doing professionally. Intervention in role-play focused on the client's ability to respond to such questions. Discussions were also held with the supervisor and the client’s parents about the action pathways for the client’s future goals.

While the period followed by gifting was marked by better therapeutic alliance and engagement in therapy, not all the effects were entirely positive. The client expressed increased emotional attachment to the therapist after the gift. She stated that her fears about losing the current therapist were reinstated. Additionally, her tendency to ask the therapist personal questions increased, and a push-pull dynamic was seen in her interactions with the therapist. These “side effects” had been anticipated in the course of psychotherapy and were part of the case formulation, irrespective of the gifting. Nevertheless, these feelings were managed in the therapy like any other alliance event and were not brought up by the client after the initial sessions following the gift-giving.

A few weeks later, the client expressed difficulties with motivation and a desire to ask why she was being asked to do a lot of work in the sessions. The sessions then refocused a few minutes on matters important to the client such as learning new words in English or how to operate a laptop, besides the main area of intervention. This untangible gift of time was seen to act as a reinforcement for the client. 

It had been planned that the gifting could be repeated three months after the first gift in case of the resurgence of alliance difficulties. The second gift was given about four to five months after the first gift, when a shift was observed in the client’s motivation and engagement in therapy. Overall, after gifting, significant engagement in therapy was noted with better reports as well as observation of outcomes in terms of social and occupational functioning. The treating team was able to complete the planned therapeutic activities and a reasonable improvement in the client’s functioning levels. In turn, symptom relapses were reduced, and the client was seen to manage and prevent further breakdowns. Additionally, none of the episodes of paranoia in the therapeutic relationship were repeated. As therapy approached termination, the client expressed gratitude by gifting the therapist a handmade greeting card. At follow-up a year later, the client and the family reported sustained improvements in socio-occupational functioning, suggesting that the therapeutic gains were likely maintained.

Reflections of the trainee therapist

Initially, in psychotherapy, the trainee therapist started with a problem-solving approach. The enthusiastic trainee therapist wanted to dive in and start her work using her limited cognitive-behavioural therapy knowledge. The supervisor enquired into the rationale for choosing CBT and the trainee’s idea of the client’s cognitive capacities. The trainee found it challenging to understand that the client had any cognitive limitations, insisting that the client was just as capable as anyone else of reflecting on her own thoughts and emotions. The beginner therapist experienced significant difficulty in assessing the client’s available resources as opposed to her aspirations. However, as therapy progressed, the client’s difficulties in abstract reasoning and social cognition became apparent. This gave the trainee the space to engage in further reflection on the trainee therapist’s need for the client to be amenable to in-depth work and discussion. An elaborate supervisory discussion was held on defining and redefining what therapy means for the trainee therapist. Ideas of doing some structured, elaborate, or intellectual work that could change the lives of individuals were uncovered, and supervisory discussions focused on reflecting on these ideas. This was followed by supportive work as the client reported emotional disturbances due to familial conflicts and expressed emotions.

The trainee therapists' initial reactions and thoughts were marked by a sense of shock and discomfort when the supervisor suggested the idea of gifting the client. This discomfort was exacerbated by the therapists' concern about potentially losing the sense of boundaries in the relationship, particularly given the client's proclivity for asking personal questions and commenting on the dressing style of the therapist. The therapist feared that this strategy could go terribly wrong, as she felt overwhelmed enough by the other challenges and the suggestion of seeing her first therapy client three times a week. Even when buying their gift, the therapist considered various options anxiously. However, the therapist found that she could manage her anxieties about speaking to clients in general after repeated sessions of this intensive nature with a “difficult client,” making it an enriching training experience with hands-on learning.

The trainee therapist was able to navigate the initial anxiety and present her first gift to the client. The therapist was much more comfortable with the observation of increased engagement in therapy post-gifting. Nevertheless, some concerns that the therapist harboured did materialize. There was an observable increase in the frequency of personal questions directed towards the therapist and expressed fears of becoming too attached to or too reliant on the therapist. In her fear of developing countertransference, the therapist also felt anxious when she found herself imagining herself as the client’s family member in the session when the client recounted some of the occurrences in the family. She experienced feelings of guilt as if she was doing something wrong by engaging with a client in this manner and brought these concerns to the supervisor’s notice.

The therapist acknowledged the client’s feelings, and the supervisor acknowledged the therapist’s feelings. These “concerning behaviours” of increased interest in the therapist and the clients, feared to be transferential, were eventually considered helpful signs of the client's engagement with the world around her and with the therapist. In any case, the trainee therapist, though laden with doubts, was able to argue that there was no unmanageable dependence or side-effect of gift-giving, and the outcomes of therapy were also positive, including at one-year follow-up. Further, the trainee therapist reflected on the cognitive change with respect to her beliefs about ethics in psychotherapy, understanding that it was the personal responsibility of the therapist to abide by the ethical principles and the therapeutic plan with clear motives and thoughts.

## Discussion

The empirical silence on gifting is alarming despite the finding that both helpful and unhelpful gifting episodes ultimately facilitate the therapeutic process [4]. Ethical caution prevails in the scholarly and clinical discussions on gifting. On the other hand, there is a growing recognition of the importance of therapist presence and responsiveness in psychotherapy [18]. Responding to each client's unique needs and challenges across various time points in therapy may enhance the quality of the bond shared by the client and the therapist. Increased efforts from the therapist’s side have also been linked to better client engagement in psychotherapy [19]. In light of this literature, it may be necessary to reconsider therapists' extremely cautious approach regarding boundaries and ethics.

In the current case study, the client’s ability to form abstract thoughts and interpersonal relationships was seen to be impaired, along with other challenges elaborated on in the earlier sections. The burden of conveying to the client that they are valued and that the therapists are engaged then fell on the therapeutic team. Sometimes, the alliance may need more tangible forms of strengthening, especially with children or adult clients in the concrete-operational mode of functioning. Thus, while the act of gifting in itself is concrete, symbolic meanings are attached to it. To ensure that the symbolic meaning is conveyed well and not misinterpreted, therapists must be cautious in the way they gift or receive gifts.

The context here in our case study is important - the client reported very few social interactions outside her family and a lack of stable friendships. She was always appreciative of the accessories worn by others. As an attempt to respond to the client’s way of relating with the therapist and others, the gift was carefully tailored to be an accessory (earrings) for the client to wear. Our motive was in line with the client's formulation, and there was a clear expectation of improved therapeutic outcomes. It is important to note that the gift was inexpensive and small in nature. As mentioned earlier, the treatment setting was a public hospital with minimum or no fees for clients, depending on their socio-economic strata. As for the cultural context, even within the Indian culture, there are different narratives on gift-giving, depending on the subculture involved. The Bhagavad Gita, for instance, condemns receiving favours from others but encourages donating gifts to others [20]. However, exchanging gifts during festivals is quite common and historically prevalent. In some subcultures in India, gifts are exchanged in a very transactional manner, with the tendency to return gifts of a similar value to ones that are received [20]. The cultural appropriateness of the gift is also a key factor in determining the appropriateness of the gifting process. Giving and receiving gifts in some parts of Northern India is generally considered a periodic phenomenon in any relationship, symbolic of the individual’s appreciation for the person. The client’s interpretation of the gift was also seen to be culturally appropriate, as she appreciated the earrings gift as a “good gesture” from the therapist.

The discussion is incomplete without touching on the potential problems that did or could have arisen in the psychotherapy process in the case. A sense of obligation or undue influence may be exerted on the client, where they may feel compelled to gift the therapist back [12]. While such matters may be managed through direct and effective communication, the client may still feel like they owe the therapist, which may result in pressure and potentially disrupt client autonomy. The newer literature on such undue influence in the context of research has indicated that such influences may be rare and only harmful if they cause the client to unsee the risks of participating in a study [21]. Glass [21,22] productively clarifies the difference between boundary crossing and boundary violation as described by Gutheil and Gabbard [16] and further prompts clinicians to move beyond the polarized categorization of ethical greyness. It would be interesting for clinicians to assess the validity of these claims in their practice and research settings.

On the other hand, the clients may feel more attached to or dependent on the therapist, which may pose a threat to their independence in the therapeutic outcomes. It may be important to consider here that such dependence may occur irrespective of gifting. It may also be considered a “side effect” to manage and tolerate, given that the main therapeutic benefits exceed such side effects. Lastly, it would also be grossly inappropriate to gift clients who may be restricted in their expressions of any negative feedback for the therapists, or, in other words, maybe “therapist-pleasers.” Therefore, assessing a client’s suitability for such an experiment and defining the indications and contraindications may be essential. As the situation may differ across clients and contacts, there is no “one size fits all” formula for gift-giving. Nevertheless, we have attempted to summarize a framework of ethical gift-giving based on the literature and our experience.

The initial anxiety experienced by the trainee therapist is in line with previous reports of anxiety in resident therapists in gifting children clients [12]. It is also in line with the archaic slippery slope argument [16], which assumes that the therapist is incapable of restricting their ethical actions. Additionally, the anxiety also probably stemmed from the lack of a developed understanding of the ethical principles of psychotherapy. It would be essential to consider the lacunae in the training and development of therapists, especially with regard to ethical dilemmas and areas of ethical greyness.

As highlighted earlier, the literature is very limited when it comes to the therapeutic use of gifting clients. The current paper reports at least one case experience with ethical gift-giving that starkly contradicts Freud’s experiences with Ratman. More studies, such as the current one, need to be implemented and published to understand the clinical phenomena and their consequences better. An open, non-stigmatized discussion of ethical transgressions as a whole is important because otherwise, the unsure therapist may take an overly cautious approach at most times, not venturing into areas considered grey or stigmatized, such as gifting.

The treating team also reflected on a variety of factors besides the gifting that could have added up to help achieve the gains. For instance, regular engagement in occupational therapy and increased compliance with medicines could have facilitated the therapeutic changes. Often, in psychotherapy, a mix of factors may lead to remission, and this must be kept in mind while interpreting the current case study. Additionally, the case study methodology has its limitations, and more process-based research in rehabilitative settings may be helpful to further clarify the gains achieved from each of the factors. The findings from one case study may not be generalizable. Nevertheless, they may help generate more research and discussion around the ethical frameworks followed in psychotherapy.

While controversial, in the current study, we observed an increase in the client’s engagement due to the gifting. The therapy outcome was also satisfactory, even with the ambitious goals set for the client in the context. The question we aim to raise is, just like self-disclosure, is limited gift-giving ethical and an intervention to be considered in cases with such complexities? More importantly, a larger question is raised about ethics in psychotherapy: what are the reasons underlying stringent ethical perceptions of psychotherapists? We have elaborated on a list of considerations that may act as a framework for any therapist who may consider experimenting with the concept of gifting the client to enhance their therapeutic alliance.

A proposed framework for ethical gift-giving in psychotherapy

First and foremost, it must be said that gifting may not be an ideal strategy to enhance alliances with many clients. For instance, if the client is romantically interested in the therapist, the gift from the therapist may be misinterpreted easily. Determining the client’s suitability for gifting is the first imperative step.

While it is clear that gifting is not always acceptable and relies heavily on context, there is also an air of caution surrounding matters of such ethical dilemmas. While safeguarding the practice of the therapist, there might be some ways to experiment with the ethical boundaries, so long as a framework is followed and documented.

Context is important. When it comes to the clinical context, while diagnosis is important [4], a therapeutic formulation is of central importance to understanding and implementing ethical gift-giving. It is important to consider questions like “What will be the potential impact of the gift on the therapeutic alliance?” A cost-benefit analysis is usually helpful in this regard. Potential costs, as highlighted, include misinterpretation of the gestures as romance, friendship, or a personal connection.

The motive of the gifting and the receiving of gifts is important to assess, as has been highlighted by multiple ethical codes. The therapeutic purpose should be clear, i.e., there should be a clear expectation of improved therapeutic alliance or outcomes. Similar to other therapeutic interventions, the gift should be mentioned in the therapeutic notes.

Socio-demographic and cultural context is also essential. The gender and sexual orientations of the client and the therapist, if known, must be considered to rule out the possibility of a romantic/intimate suggestion. The age of the client, including their developmental and cognitive abilities, must be considered. There is a wider acceptance of receiving gifts from children in the concrete operation stage, for instance [9]. Cultural connotations and practices are also important to consider and, therefore, to be aware of or sensitive to.

The nature and monetary value of gifts. The value of the gift must not exceed a certain amount and should be inexpensive. Gifts with a possible intimate, romantic, or sexual connotation, like flowers, should be avoided. Handmade and personalized gifts are considered more acceptable in general. Edibles are considered more acceptable in general, probably because they are inexpensive, perishable, and culturally sanctioned. Gifts that enhance the therapy process, such as books on the subject matter, may also be considered appropriate.

Generally, the beginning of therapy is considered a bad time for gifting, and special occasions or certain achievements are considered good times in play therapy literature [4,9]. However, the rationale may justify gifting during the middle stages of therapy, as in our case study.

The gifting should not be too frequent [9] but should be decided a priori with a clear rationale.

The assessment of the client's understanding of receiving (or giving) the gift can be conducted through observation or interviewing, using questions such as "How do you feel about the gift I gave you?" Knox [4] recommends undertaking such an assessment both in the short and the long term.

Supervision and consultation are strongly recommended when it comes to matters of ethical dilemmas, such as gifting, to receive an objective perspective on the appropriateness of the activity [9].

The decision-making process incorporates ethical principles.

Have an open discussion with clients about gifting, and include terms related to gift-giving and receiving in informed consent forms [9].

## Conclusions

In the current paper, we set out to describe a case study with a complex presentation of chronic schizophrenia and difficulties in alliance formation. Our review highlighted an extreme cautionary stance against gifting for therapeutic purposes, especially when working with adult populations. This stance seems outdated and conservative, with roots in psychoanalytic case studies and arguments of slippery slopes in ethics. We highlight the need to distinguish between ethical boundary violations and boundary crossings in psychotherapy.

We sought to reflect on and challenge the cautious approach, with the help of the current case in which we experimented with gifting the client non-valuable, small objects to strengthen the therapeutic alliance. The positive outcomes of such a process, both in terms of alliance formation and therapeutic gains, are encouraging but also warrant more in-depth analysis and process-based objective research. We also summarized the available literature and guidelines for ethical considerations in gifting a client, proposing a framework for practitioners to apply. Key pointers include the clarification of the therapeutic motive and therapeutic formulation behind the gifting, client suitability, and consideration of the culture and context of the therapeutic work.
